# The association between A Body Shape Index and mortality: Results from an Australian cohort

**DOI:** 10.1371/journal.pone.0181244

**Published:** 2017-07-31

**Authors:** Janet F. Grant, Catherine R. Chittleborough, Zumin Shi, Anne W. Taylor

**Affiliations:** 1 School of Public Health and Robinson Research Institute, The University of Adelaide, Adelaide, South Australia, Australia; 2 Population Research and Outcome Studies, Discipline of Medicine, The University of Adelaide, Adelaide, South Australia, Australia; Medical University of Vienna, AUSTRIA

## Abstract

It is well recognised that obesity increases the risk of premature death. A Body Shape Index (ABSI) is a formula that uses waist circumference (WC), body mass index (BMI) and height to predict risk of premature mortality, where a high score (Quartile 4) indicates that a person’s WC is more than expected given their height and weight. Our study examines the association between ABSI quartiles and all-cause-, cardiovascular- and cancer-related mortality, and primary cause of death. Self-reported demographic and biomedically measured health-related risk factor and weight data was from the baseline stage of the North West Adelaide Health Study (1999–2003, n = 4056), a longitudinal cohort of Australian adults. Death-related information was obtained from the National Death Index. Primary cause of death across ABSI quartiles was examined. The association between mortality and ABSI (quartile and continuous scores) was investigated using a Cox proportional hazards survival model and adjusting for socioeconomic, and self-reported and biomedical risk factors. The proportion of all three types of mortality steadily increased from ABSI Quartile 1 through to Quartile 4. After adjusting for demographic and health-related risk factors, the risk of all-cause mortality was higher for people in ABSI Quartile 4 (HR 2.64, 95% CI 01.56–4.47), and ABSI Quartile 3 (HR 1.95, 95% CI 1.15–3.33), with a moderate association for the continuous ABSI score (HR 1.32, 95% CI 1.18–1.48). ABSI is therefore positively associated with mortality in Australian adults. Different combined measures of obesity such as the ABSI are useful in examining mortality risk.

## Introduction

It is well recognised that obesity increases the risk of premature death. A number of studies from the United States (US), but also from studies based in Europe, Asia and Australia, have examined the link between obesity and mortality based on a range of individual anthropometric measures including body mass index (BMI)[1–5], waist circumference (WC)[6], waist hip ratio (WHR)[7], together with studies that have combined all or some of these measures[8–10] with the addition of skinfold measures[11] and waist thigh ratio (WTR)[12].

Regardless of which obesity measure was used, there was a general consensus that being overweight and particularly obese, increased the risk for premature death from all-causes, and for cardiovascular (CVD) conditions and cancer. Wang et al [13] report that for each additional 5kg/m^2^ in BMI, there is a subsequent increased risk of developing oesophageal (52%) and colon (24%) cancer for men, and for endometrial (59%), gall bladder (59%) and postmenopausal breast cancer (12%) for women. In their study of the projected health and economic burden of obesity for 2030 in the US and the United Kingdom (UK), they estimate that 2.1-2.4 million (US)/179,000-230,000 (UK) incident cases of diabetes, 1.4-1.7 million (US)/122,000 (UK) incident cases of cardiovascular disease and 73,000-127,000 (US)/32,000-33,000 (UK) incident cases of cancer may be preventable, if adults were to reduce their BMI by 1% [13].

Similar results may be possible in Australia, due to the similarity of primary causes of death. Nine of the twenty leading causes of death for 2015 for Australians were obesity-related, namely (in order) ischaemic heart disease (1), cerebrovascular diseases (3), diabetes (6), colon and associated cancers (7), heart failure (9), breast cancer (14), pancreatic cancer (15), cardiac arrhythmias (17), and hypertensive diseases (18)[14].

Studies based on BMI highlighted the ‘obesity paradox’, finding concave associations of people with both lower and higher BMIs having lower survival rates than those in the normal-overweight BMI range.[5,15,16] These findings have been countered by other studies that have highlighted the limitations of using BMI in predicting mortality due to its inability to measure central adiposity or to distinguish between fat mass and lean body mass. BMI has undetermined validity for use as a measure of fatness in older people (as aging is generally associated with a considerable loss in lean body mass and some increase in fat mass). Further, a low BMI may result from one or more underlying issues, possibly distorting the relationship between body shape and premature mortality. An additional element is the generally lower body weight of smokers who consequently have higher rates of premature mortality.[6,17,18] The importance of including measures of central adiposity were emphasised in a recent study of all-cause and CVD-related mortality risk among people who had normal weight but with central obesity, which found twice the mortality risk of those who were overweight or obese based on BMI alone[19].

In recognition of the need to incorporate a measure of central adiposity in a formula that could more accurately predict mortality risk than using BMI alone, *A Body Shape Index* (ABSI) was developed.[20] This index is based on a person’s WC whilst adjusting for their height and weight.[21] The ABSI was developed to examine the mortality hazard and characteristics of populations, and its predictive power was proven to be consistent over a minimum period of 20 years[22].

The ABSI is relatively new and therefore studies are still emerging regarding different aspects of this measure, including differences between males and females. A recent study validated ABSI for its predictive power for total and cause-specific mortality in comparison with BMI, WC, WHtR and WHR in a middle-aged population of 2626 men and 3740 women from the Netherlands, and found ABSI to have a stronger association than the other weight measures but with limited added predictive value. It also found a stronger association with mortality with a HR per 1 SD increase in ABSI of 1.15 for men and 1.10 for women (95% CIs 1.08–1.29 and 0.99–1.22 respectively)[21].

During a review of literature regarding the ABSI, it was observed that no study had yet explored the cause of death for those within each ABSI quartile. Our study provides these characteristics, as well as assessing the predictive power for mortality of ABSI using both quartiles and the continuous score for all-cause, CVD-and cancer-related mortality in an Australian population.

## Material and methods

### Sample

The North West Adelaide Health Study (NWAHS) is a longitudinal study of 4056 randomly selected adults aged 18 years and over recruited from the north-west region of Adelaide, the capital of South Australia. All households in the northern and western areas of Adelaide with a telephone connected and a telephone number listed in the Electronic White Pages (EWP) were eligible for selection in the study. Participants were recruited from 1999 to 2003 through an initial Computer Assisted Telephone Interview (CATI), and the eligible adult to have had the most recent birthday in the household was invited to participate. Respondents were excluded if they did not have the capacity to participate due to illness or intellectual limitations, together with those who were unable to communicate in English and those living in a residential institution. Data collection incorporated questions in the CATI survey and a self-completed questionnaire, as well as a biomedical examination that included anthropometric measures. The study methodology has been previously described in detail.[23,24] Our study was conducted according to the guidelines laid down in the Declaration of Helsinki and all procedures involving human subjects/patients were approved by the Human Research Ethics Committee of The University of Adelaide and of the Central Northern Adelaide Health Service (The Queen Elizabeth and Lyell McEwin Hospitals). Written informed consent was obtained from all participants.

Data used in this paper are drawn from baseline recruitment (Stage 1, n = 4056, response rate 49.1%). Characteristics of the study participants at baseline, as well as a comparison with Australian Census and local data, have been published elsewhere.[25] The main analysis sample (n = 3311) comprised those participants with complete data available for those variables included in the model, including those participants who died between 2000 and 2015. Missing values were not imputed due to very few differences being observed between the baseline and analysis samples.

#### Mortality

Overall, 581 participants (14.3% of the original cohort) died between January 2000 and September 2015. There are four administrative levels regarding notification and confirmation of cohort participant deaths. Of the 581 deaths, 207 deaths had minimal information available for analysis purposes. The first level related to the most recent deaths (n = 32), which had been communicated by family or friends to the cohort study co-ordinator, but were yet to be confirmed by the South Australian Births, Deaths & Marriages Office. The second level related to a number of deaths of which the study had been notified within the past year, which were subsequently confirmed by this authority (n = 104). The third level involved a process where demographic data of both those participants who were being tracked as well as notified deaths (from family and friends, as well as notifications from the registry of South Australian Births, Deaths and Marriages) are submitted on an annual basis to the National Death Index (NDI), and matched using a probabilistic record-linking software with the National Mortality Database (NMD), facilitated by the NDI which is maintained by the Australian Institute of Health and Welfare, and the National Coronial Information Service (NCIS). The NDI uses multiple passes that incorporates full names, sex, dates of birth, last contact and death, with the Australian State at their last known address in a weighted algorithm that identifies matches between NDI records and study participants. Date of death was only available from the NDI for the more recent deaths (from 2013 onwards, n = 71). For our study, the date of last contact was used if the date of death was unable to be determined. There is at least a two year time lag following preparation and submission of cohort participant details to the NDI regarding cause of death information; this was subsequently provided on 374 participants at the fourth and final level (from January 2000 to December 2012). One primary and up to seven secondary causes of death were supplied using the 10th revision of the *International classification of diseases* (ICD10). Cardiovascular disease (CVD)-related deaths were classified as ICD10 codes of I00 to I99 (diseases of the circulatory system), while cancer-related deaths were classified as ICD10 codes of C00 to D49 (neoplasms).

#### A Body Shape Index

The ABSI was developed as an indication of risk that incorporates the excess risk of WC while adjusting for BMI and height[20]. The ABSI was based on baseline data on non-pregnant adults aged 18 years and over (n = 14,105) from the 1999–2004 US National Health and Nutrition Examination Survey (NHANES), and was evaluated for prediction of mortality using the US National Death Index data through to December 2006 (2–8 years of follow-up; average 5 years (828 deaths).[20] Krakauer and Krakauer[20] performed liner least-squares regression on log(WC) as a function of log (height) and log (weight) for the sample, and then approximated the obtained regression coefficients with ratios of small integers (WC ∞ weight^2/3 height-5/6^) to produce the final formula (WC / BMI^2/3^x height^1/2)^, A z score can also be calculated from the ABSI mean and standard deviation (SD) within a population, dependent upon adjustment for age and sex (ABSI-ABSI_mean_/ABSI_SC_). Online calculators are available that calculate an ABSI value and the z score, and then provide information for comparison purposes for a person of the same sex and age, as well as an indication of individual mortality risk via quintiles (also see https://nirkrakauer.net/sw/absi-calculator.html that provides relative risk values for BMI and ABSI). For the purposes of this paper, the ABSI continuous score was classified into quartiles; Quartile 1 being the lowest and Quartile 4 being the highest. A high ABSI indicates that a person's WC is more than expected, given their height and weight, corresponding to a higher concentration of body volume centrally[20].

#### Body shape measures

Height without shoes was measured to the nearest 0.5 centimetres using a wall-mounted stadiometer and weight to the nearest 0.1 kilogram in light clothing and without shoes using standard digital scales. BMI was calculated by dividing the participant's weight in kilograms by the square of their height in metres (kg/m^2^). WC was measured to the nearest 0.1 centimetre using an inelastic tape maintained in a horizontal plane, with the subject standing comfortably with their weight distributed evenly on both feet. The measurement was taken at the level of the narrowest part of the waist, and the mean calculated from three measurements of the waist. A high WC was defined as being at least 102 cm for males and 88 cm for females[26].

#### Risk factors

A number of risk factors were included in this research. Participants were asked in a questionnaire if they currently smoked or if they had ever smoked regularly (at least once a day) and their responses were categorised into non-smokers, ex-smokers and current smokers. Alcohol risk was based on the amount and frequency of alcohol usually consumed, and categorised into non-drinkers and no-, low-, intermediate-, high- and very high risk drinkers.[27] Participants were also asked nine questions that comprise the physical activity component of the National Health Survey[28] and their results were calculated on the formula "e × t × i"" where e was number of times walking, moderate and/or vigorous exercise was undertaken during the past two weeks, t was the average amount of time spent on each exercise session and i was the intensity (walking scored at 3.5, moderate exercise scored at 5.0 or vigorous exercise scored at 7.5). Participants were classified as sedentary (score less than 100, including no exercise), or as having low (score of at least 100 but less than 1600), moderate (score of at least 1600 to 3200, or more than 3200 but less than 2 hours of vigorous exercise) or high (score of at least 3200 and 2 hours or more of vigorous exercise) levels of physical activity. This risk factor was further reduced to a dichotomous variable–sedentary or undertaking some level of physical activity[29].

Clinic attendees had their blood pressure measured using a standard, calibrated blood pressure sphygmomanometer. Two blood pressure measurements were taken five to ten minutes apart while the participant was relaxed and seated, and the average calculated. From these, a variable was derived classifying high blood pressure as at least 140/90 mmHg[29] (systolic and/or diastolic). A fasting blood sample of approximately 10 ml was taken for a number of blood-related measures, and the results were dichotomised according to recognised cut-off values, including total blood cholesterol[30](< or ≥5.5 mmol/L), triglycerides[31] (< or ≥ 1.7 mmol/L), glycated haemoglobin[32] (HbA1c) (< or > 7%). Study participants were also asked if they had a parental history of diabetes, heart disease and/or stroke.

#### Demographics

Demographic variables at Stage 1 included age, sex, marital status, work status, gross annual household income (before tax deducted), highest educational qualification achieved and country of birth.

### Statistical analysis

The unweighted data were initially analysed using SPSS Version 20.0 (IBM, Armonk, NY) and the final analysis used Cox regression using Stata Version 13 (StataCorp, College Station, TX). Univariable analyses were undertaken on weight measures, mortality-related variables, together with demographic and health-related risk factors at baseline for the overall cohort and the analysis sample. The mean, standard deviations and p values were calculated for age, BMI and WC, together with the proportions, number and p values for all-cause-, CVD- and cancer-related mortality across ABSI quartiles; analyses are provided for overall, as well as male and female. Person years were calculated from the date of the baseline biomedical clinic appointment, and either the date of death or date of last contact for use in a Cox regression. The overall association between all-cause mortality and ABSI quartiles, BMI categories and high WC categories were examined using a Cox proportional hazards survival model to examine these measures as predictors of all-cause mortality (CVD- and cancer-related mortality and differences between males and females could not be explored due to small numbers in the lower quartiles). ABSI Quartile 1 (lowest BMI/WC), BMI<18.5 (underweight) and WC of <94cm (males) and <80cm (females) were the reference categories and the hazard ratio (HR), and the relative risk (RR), 95% confidence intervals and p value for each is provided. The first model adjusted for age and sex; while the second model adjusted for age, sex, demographic characteristics (marital status, work status, annual gross household income, highest educational qualification achieved, country of birth) and health-related risk factors (smoking, alcohol risk, physical activity level, high blood pressure, high total blood cholesterol, high triglycerides, high glycated haemoglobin, and parental history of disease-diabetes, heart disease and stroke). A Kaplan-Meier survival graph was used to show the differences in survival by ABSI quartiles.

## Results

An overview of selected demographic, socioeconomic and health-related risk factor characteristics for study participants at baseline for the original response sample (n = 4056) versus the analysis sample for the hazard ratios (n = 3311) is shown in Table 1. There were minimal differences between the characteristics of each sample. Table 1 shows that within the analysis sample, 12.9% of participants had died (all-causes), including 4.3% from cardiovascular-related causes and 3.6% from cancer-related causes.

**Table 1:** Descriptive variables for baseline original response sample and analysis sample (unweighted).

	*Original sample* *(n = 4056)*			*Analysis sample* *(n = 3311)*			
* *	*n*	*%*	*Mean (SD)*	*n*	*%*	*Mean (SD)*	*P value*
**WEIGHT MEASURES**							
**BMI (n, mean, SD)**	**4054**		27.8 (5.5)	3311		27.8 (5.4)	0.712
**BMI (n, %)**							0.899
Underweight <18.50	44	1.1		33	1.0		
Normal 18.50–24.99	1289	31.8		1047	31.6		
Overweight 25.00–29.99	1562	38.5		1301	39.3		
Obese 30.00+	1159	28.6		930	28.1		
**WC (cm) (n, mean, SD)**	**4053**		92.5 (14.7)	**3311**		92.3 (14.5)	0.600
**WC (cm) (n, %)**							0.753
Normal WC (M<94cm, F<80 cm)	1427	35.2		1175	35.5		
Overweight WC (M94-101cm, F80-87cm)	1005	24.8		839	25.3		
Obese WC (M> = 102cm, F> = 88cm)	1621	40.0		1297	39.2		
**ABSI**							0.862
Quartile 1	1013	25.0		840	25.4		
Quartile 2	1013	25.0		835	25.2		
Quartile 3	1013	25.0		837	25.3		
Quartile 4	1013	25.0		799	24.1		
**MORTALITY**							
All-cause	581	14.3		427	12.9		0.076
CVD-related*	209	5.2		143	4.3		0.095
Cancer-related *	157	3.9		120	3.6		0.580
**DEMOGRAPHICS**							
**Age**							0.277
20 to 34 years	755	18.6		626	18.9		
35 to 54 years	1670	41.2		1414	42.7		
55 to 74 years	1275	31.4		1015	30.7		
75 years and over	356	8.8		256	7.7		
**Sex**							0.839
Male	1932	47.6		1585	47.9		
Female	2124	52.4		1726	52.1		
**Marital status**							0.338
Married/defacto	2461	60.7		2068	62.5		
Separated/divorced	579	14.3		471	14.2		
Widowed	375	9.2		270	8.2		
Never married	618	15.2		502	15.2		
**Work status**							0.370
Full time employed	1431	35.3		1246	37.6		
Part time/casual employed	690	17.0		582	17.6		
Unemployed	146	3.6		114	3.4		
Home duties/retired	1520	37.5		1191	36.0		
Student/other	226	5.6		178	5.4		
**Annual gross household Income**							0.447
Up to $20,000	1193	29.4		977	29.5		
$20,001 to $40,000	1029	25.4		896	27.1		
$40,001 to $60,000	799	19.7		708	21.4		
$60,001 and over	806	19.9		730	22.0		
**Highest education level**							0.302
Secondary	1749	43.1		1432	43.2		
Trade/Apprentice/Certificate/ Diploma	1686	41.6		1446	43.7		
Bachelor degree or higher	473	11.7		433	13.1		
**Country of birth**							0.864
Australia	2777	68.5		2291	69.2		
United Kingdom/Ireland	700	17.3		584	17.6		
Europe	394	9.7		310	9.4		
Asia/other	164	4.0		126	3.8		
**RISK FACTORS**							
**Smoking**							0.782
Non-smoker	1819	45.1		1487	44.9		
Ex-smoker	1321	32.8		1108	33.5		
Current smoker	892	22.1		716	21.6		
**Alcohol**							0.607
Non-drinker/no risk	2152	53.5		1733	52.3		
Low risk	1648	41.0		1393	42.1		
Intermediate to very high risk	223	5.5		185	5.6		
**Physical activity**							0.555
Sedentary	1035	28.2		912	27.5		
Undertakes some form of exercise	2638	71.8		2399	72.5		
**High blood pressure** (≥140/90 mmHg)	1253	30.9		986	29.8		0.302
**High total blood cholesterol** (≥5.5 mmol/L)	1580	39.4		1298	39.2		0.842
**High triglycerides** (≥1.7 mmol/L)	1128	28.2		927	28.0		0.885
**High HbA1c** (>7%)	140	3.5		101	3.1		0.287
**PARENTAL HISTORY OF DISEASE**							
**Diabetes**	745	18.4		612	18.5		0.898
**Heart disease**	1507	37.2		1228	37.1		0.953
**Stroke**	798	19.7		650	19.6		0.963

Note: Not stated not shown

* Either a primary or subsequent cause of death

Table 2 shows the mean and standard deviation for overall and then by sex for age, BMI and WC for each ABSI quartile at baseline, as well as the proportion and number of those participants with a high WC, and those who have subsequently died from all-cause, CVD-related and cancer-related mortality. P values are provided for males and females within each quartile, and across all quartiles. Overall, mean age increased across the quartiles from 42.0 years in Quartile 1 (31.1/43.2 males/females) to 60.4 years in Quartile 4 (59.6/63.1 males/females). Mean BMI remained in the overweight range (25–29) for all ABSI quartiles overall, however WC steadily increased and the proportion of people with a high WC increased from 21.7% in Quartile 1 to 58.5% in Quartile 4, with females more likely than males to have a high WC. The proportion of all three types of mortality examined steadily increased from ABSI Quartile 1 through to Quartile 4. Those in Quartile 4 had a higher proportion of all-cause, CVD- and cancer-related mortality than the other three quartiles combined, with a higher proportion of females than males for all mortality causes across all quartiles, except for CVD- and cancer-related mortality. The mean time between the baseline biomedical clinic appointment date and date of death was 7.5 years (range 2 weeks to 14 years).

**Table 2:** Baseline mean, standard deviation (SD) and p value for age and weight measures; proportion, n and p value for all-cause, CVD-related and cancer-related mortality across ABSI quartiles for overall, males and females (n = 4052).

*ABSI Quartiles*			*WEIGHT MEASURES*						*MORTALITY*					
	*Age (yrs)*		*BMI*		*WC (cm)*		High WC*		*All cause*		CVD- related**		Cancer- related**	
	*Mean (SD)*		*Mean (SD)*		*Mean (SD)*		*% (n)*		*% (n)*		*% (n)*		*% (n)*	
**OVERALL (n = 4052)**	**50.3**	(16.3)	**27.8**	(5.5)	**92.5**	(14.7)	**40.0**	(1621)	**14.3**	(581)	**5.2**	(209)	**3.9**	(157)
Males (n = 1932)	50.6	(16.8)	27.9	(4.8)	98.4	(13.0)	36.3	(701)	**17.7**	(341)	**6.7**	(129)	**5.2**	(101)
Females (n = 2124)	50.1	(16.1)	27.7	(6.0)	87.1	(14.1)	43.4	(920)	**11.3**	(240)	**3.8**	(80)	**2.6**	(56)
*p (ABSI quartiles)*	*<0*.*001*		*<0*.*001*		*<0*.*001*		*<0*.*001*		*<0*.*001*		*<0*.*001*		*<0*.*001*	
**QUARTILE 1 (n = 1013)**	***42*.*0***	***(14*.*2)***	***27*.*0***	***(5*.*9)***	***80*.*8***	***(11*.*8)***	**21.7**	**(220)**	**3.3**	**(33)**	**1.1**	**(11)**	**1.2**	**(12)**
Males (n = 103)	*31*.*1*	*(11*.*2)*	*25*.*4*	*(3*.*5)*	*82*.*5*	*(8*.*4)*	1.9	(2)	*-*	(n<5)	*-*	(n<5)	*-*	(n<5)
Females (n = 910)	*43*.*2*	*(14*.*0)*	*27*.*2*	*(6*.*1)*	*80*.*6*	*(12*.*1)*	24.0	(218)	3.3	(30)	1.1	(10)	1.2	(11)
*p (males/females)*	*<0*.*001*	* *	*0*.*002*	* *	*0*.*126*	* *	*<0*.*001*	* *	*-*	* *	-	* *	*-*	* *
**QUARTILE 2 (n = 1013)**	***47*.*1***	***(15*.*4)***	***27*.*6***	***(5*.*6)***	***89*.*2***	***(12*.*0)***	**33.9**	**(343)**	**7.9**	**(80)**	**2.1**	**(21)**	**1.8**	**(18)**
Males (n = 372)	*39*.*6*	*(13*.*2)*	*27*.*3*	*(4*.*7)*	*91*.*5*	*(10*.*5)*	15.9	(59)	5.1	(19)	-	(n<5)	*-*	(n<5)
Females (n = 641)	*51*.*4*	*(15*.*0)*	*27*.*7*	*(6*.*0)*	*87*.*9*	*(12*.*6)*	44.3	(284)	9.5	(61)	3.0	(19)	2.5	(16)
*p (males/females)*	*<0*.*001*	* *	*0*.*228*	* *	*<0*.*001*	* *	*<0*.*001*	* *	*0*.*012*	* *	*0*.*009*	* *	*0*.*38*	* *
**QUARTILE 3 (n = 1013)**	***51*.*9***	***(15*.*2)***	***28*.*3***	***(5*.*0)***	***96*.*7***	***(11*.*5)***	**45.8**	**(464)**	**14.6**	**(148)**	**5.0**	**(51)**	**3.6**	**(36)**
Males (n = 656)	*48*.*9*	*(14*.*4)*	*28*.*1*	*(4*.*5)*	*97*.*8*	*(10*.*6)*	32.8	(215)	11.1	(73)	3.8	(25)	2.7	(18)
Females (n = 357)	*57*.*6*	*(15*.*1)*	*28*.*8*	*(5*.*8)*	*94*.*7*	*(12*.*8)*	69.7	(249)	21.1	(75)	7.3	(26)	5.0	(18)
*p (males/females)*	*<0*.*001*	* *	*0*.*041*	* *	*<0*.*001*	* *	*<0*.*001*	* *	*<0*.*001*	* *	*0*.*016*	* *	*0*.*059*	* *
**QUARTILE 4 (n = 1013)**	***60*.*4***	***(15*.*1)***	***28*.*2***	***(5*.*2)***	***103*.*0***	***(13*.*3)***	**58.5**	**(593)**	**31.5**	**(319)**	**12.3**	**(125)**	**9.0**	**(91)**
Males (n = 800)	*59*.*6*	*(15*.*1)*	*28*.*3*	*(5*.*0)*	*104*.*1*	*(13*.*0)*	53.1	(425)	30.6	(245)	12.5	(100)	10.0	(80)
Females (n = 213)	*63*.*1*	*(14*.*9)*	*28*.*1*	*(5*.*9)*	*99*.*2*	*(13*.*7)*	78.9	(168)	34.7	(74)	11.7	(25)	5.2	(11)
*p (males/females)*	*0*.*003*	* *	*0*.*797*	* *	*<0*.*001*	* *	*<0*.*001*	* *	*0*.*250*	* *	*0*.*764*	* *	*0*.*028*	* *

* Males ≥102cm; Females ≥88cm

** Either a primary or subsequent cause of death

Table 3 shows that the association between the ABSI (quartile and continuous scores) and mortality was attenuated after adjusting for selected demographics and health-related risk factors, but was still present. Stratification by sex was not possible due to small numbers. This analyses shows that after adjusting for sociodemographic and health-related risk factors, the risk of all-cause mortality for ABSI Quartile 3 showed a strong association (HR 1.97, 95% CI 1.16–3.43, p = 0.014), increasing for ABSI Quartile 4 (HR 2.90, 95% CI 1.72–4.88, p<0.001), with a moderate association for the continuous ABSI score (HR 1.32, 95% CI 1.18–1.48, p<0.001). The risk of all-cause mortality as measured by BMI and WC was less evident.

**Table 3:** Hazard ratios (95% confidence intervals) for all-cause mortality by measures of adiposity (n = 3311).

*Weight Measure*	*Model 1*			*Model 2*		
	*HR*	*95% CI*	*p*	*HR*	*95% CI*	*p*
**BMI**						
Underweight/Normal (<24.99) (Reference)	**1.00**			**1.00**		
Overweight (25.00–29.99)	**0.75**	(0.59–0.95)	0.015	**0.74**	(0.58–0.94)	0.013
Obese (≥30.00)	**1.06**	(0.82–1.36)	0.662	**0.92**	(0.70–1.20)	0.543
**Continuous**	**1.00**	(0.98–1.03)	0.662	**0.99**	(0.97–1.01)	0.333
**WC**						
Normal WC M<94cm, F<80 cm (Reference)	**1.00**			**1.00**		
Overweight WC M94-101cm, F80-87cm	**0.95**	(0.72–1.25)	0.712	**0.87**	(0.66–1.15)	0.337
Obese WC M> = 102cm, F> = 88cm	**1.26**	(0.99–1.60)	0.066	**1.06**	(0.82–1.38)	0.639
**Continuous**	**1.01**	(1.00–1.02)	0.007	**1.00**	(1.00–1.01)	0.337
**ABSI**						
Quartile 1 (Reference)	**1.00**			**1.00**		
Quartile 2	**1.43**	(0.81–2.50)	0.215	**1.50**	(0.85–2.64)	0.158
Quartile 3	**1.97**	(1.16–3.34)	0.012	**1.95**	(1.15–3.33)	0.014
Quartile 4	**2.90**	(1.72–4.88)	<0.001	**2.64**	(1.56–4.47)	<0.001
***Continuous***	***1*.*41***	*(1*.*26–1*.*57)*	*<0*.*001*	***1*.*32***	*(1*.*18–1*.*48)*	*<0*.*001*

Model 1—adjusted for age and sex

Model 2—adjusted for Model 1 variables, plus demographics (marital status, work status, annual gross household income, highest educational qualification achieved, country of birth) and health-related risk factors (smoking, alcohol risk, physical activity level, high blood pressure, high total blood cholesterol, high triglycerides, high glycated haemoglobin, and parental history of disease-diabetes, heart disease and stroke)

**Fig 1:**
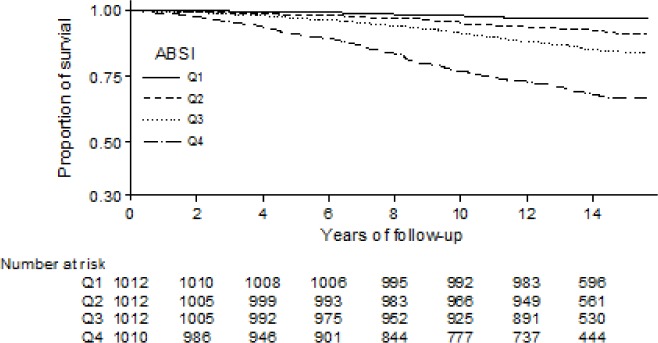
Kaplan-Meier estimate graph—ABSI quartiles.

A Kaplan-Meier estimate graph (Fig 1) provides the proportion of survival and years of follow-up by ABSI quartile. In particular, it shows an increasingly steep gradient for ABSI Quartiles 3 and 4 at approximately 0.88 and 0.65 respectively after more than 14 years of follow-up.

A review of ICD10 codes for the primary cause of death across ABSI quartiles was undertaken (n = 374) (Table 4). There was a higher proportion of deaths in Quartile 4 in the majority of cases. Supplementary information provided in S1 Table shows that this was particularly the case for malignant neoplasm of the colon (IC18.9) and the bronchus or lung (C41.0), diabetes mellitus (E14.9), acute myocardial infarction (I24.9), chronic ischaemic heart disease (I25.9), stroke (I64) and chronic obstructive pulmonary disease (J44.9).

**Table 4:** Primary cause of death by ICD10 chapters across ABSI quartiles (n = 374).

	*ABSI Quartiles*				
	*Q1*	*Q2*	*Q3*	*Q4*	*Total*
Chapter I—Certain infectious and parasitic diseases (A00-B99)	1	0	2	5	8
Chapter II Neoplasms (C00-D48)	11	17	32	80	140
Chapter III Diseases of the blood and blood-forming organs and certain disorders involving the immune mechanism (D50-D89)	0	0	0	2	2
Chapter IV Endocrine, nutritional and metabolic diseases (E00-E90)	0	1	3	10	14
Chapter V Mental and behavioural disorders (F00-F99)	0	0	1	5	6
Chapter VI Diseases of the nervous system (G00-G99)	1	1	1	3	6
Chapter IX Diseases of the circulatory system (I00-I99)	4	15	30	71	120
Chapter X Diseases of the respiratory system (J00-J99)	2	2	8	22	34
Chapter XI Diseases of the digestive system (K00-K93)	1	2	1	5	9
Chapter XIII Diseases of the musculoskeletal system and connective tissue (M00-M99)	0	0	0	2	2
Chapter XIV Diseases of the genitourinary system (N00-N99)	0	1	3	7	11
Chapter XVIII Symptoms, signs and abnormal clinical and laboratory findings, not elsewhere classified (R00-R99)	0	0	0	1	1
Chapter XX External causes of morbidity and mortality (V01-Y98)	2	4	3	12	21
**TOTAL**	**22**	**43**	**84**	**225**	**374**

Supplementary information has also been provided (S2 Table) regarding the primary and secondary/subsequent cause of death for males and females for each chapter (n = 374).

## Discussion

Our study found that those people who had the combination of the highest BMI and WC, as calculated by the ABSI, had the highest risk of premature mortality; more than two and a half times those with the lowest mortality risk. The risk of dying prematurely increased steadily across the ABSI, being one and a half times higher for those in the second quartile, to almost two times higher for those in the third quartile. Cause of death was more likely to be from CVD- and cancer-related causes, both also related to obesity.

Compared with those who had a similar BMI across the quartiles, the incorporation of the central adiposity measure found that people who had a high WC were more likely to die prematurely than those of normal WC. Females were more likely than males to have a high WC across all ABSI quartiles. They were more likely than men to be in the highest risk quartile (Quartile 4) for all-cause mortality. The importance of including central adiposity in quantifying the risk was highlighted by those who developed the ABSI measure, who found that it outperformed former standard measures of abdominal obesity including WC, waist-hip ratio (WHR) and waist-height ratio (WHtR) in predicting mortality risk. Our study also was consistent with these results regarding the greater predictability of the joint obesity measure (ABSI) compared to the single measures of obesity of BMI and WC. The developers of the ABSI found that among a British population, the risk prediction held over time (follow-up of at least 20 years) and that those whose ABSI score increased over approximately seven years subsequently had a greater mortality risk compared to those whose ABSI which had decreased.[22] In an examination of measures of body shape and their association with mortality, ABSI was also found to be the strongest predictor of all-cause mortality among an Iranian population, except for WHR in women.[21,33] Sahakyan et al[19] found that those with a normal BMI of 22 but who had central adiposity (high WHR) had a higher total mortality risk than those who had a similar BMI but no central adiposity (males HR 1.87, 95% CI 1.53 to 2.29; females HR 1.48, 95% CI 1.35–1.62). Further, these same people had higher mortality risks than those considered overweight or obesity by BMI only, particularly for males (HR 2.24, 95% CI 1.52 to 3.32; females HR 1.32, 95% CI 1.15–1.51)[19].

Our study found that males were more likely than females (32.3%) to die from CVD-related diseases. This concurred with an AusDiab study[34]that found that obese Australian males had a higher risk of myocardial infarction (hazard ratio (HR) 2.75; 95% CI 1.08–7.03) than females (HR 1.43; 95% CI 0.37–5.50). In the AusDiab study, the adverse influence of abdominal obesity was found to be associated with type 2 diabetes, dyslipidaemia, hypertension and the metabolic syndrome, with odds ratios ranging from 2 to 5, and population attributable fractions ranging from 13% to 47% with the highest proportion being for type 2 diabetes.[34] It has been reported that men who are overweight or obese at midlife were found to have an increased risk of coronary heart disease of 25% and 60% respectively.[35–40] The degree of obesity is significant in predicting mortality with a study of 1,248 Spanish study participants reporting a HR of 1.84 (95% CI 1.15–2.93) for cardiovascular plus all-cause mortality and a HR of 1.94 (95% CI 1.11–3.42) for all-cause mortality for those people with a BMI of ≥35 compared to non-obese people (BMI <30)[41].

It has been hypothesised that a developing phenotype of a metabolically healthy obese (MHO) population would have a lower risk of complications associated with obesity such as metabolic syndrome and insulin sensitivity. A review found instead that MHO was significantly associated with all-cause mortality (at 30% from two studies), CVD mortality (at 14% from one study) and incident CVD (at 33% from three studies). [42] The association with CVD mortality was further supported by four of six subclinical studies showing associations of MHO with CVD disease, such as increased carotid artery thickness and coronary artery calcium within the MHO population, compared to a metabolically healthy, normal weight population[42].

Our study used baseline weight measures and the length of time that the cohort had been overweight or obese at that time was not determined, however the time that a person lives with obesity has been shown to increase their mortality risk. The authors of a study of Framingham cohort participants reported that as the number of years living with obesity increased, so did their adjusted HR for mortality. Compared to those who were never obese, the HR ranged from 1.51 (95% CI 1.27–1.79) for those obese between 1 and 4.9 years to 2.52 (95% CI 2.08–3.06) for those living with obesity for 25 years or more. They found a dose-response relationship for all-cause, CVD- and cancer-related, as well as other-cause mortality, with each additional two years of obesity providing a HR of 10.06, 1.07, 1.03 and 1.07 respectively.[43] Further, it has been reported that a dynamic measure rather than a static measure of weight status was found to be more predictive of mortality.[35] Supporting evidence from Zheng et al.[35] found that approximately 7% of deaths after 51 years of age through to age 77 were due to obese Class I (BMI 30–34.9) and Class II/III (BMI ≥35) upward trajectories (increasing weight gain), with increases in mortality risk of 25% and 128% respectively when compared to those who were stable overweight. It has been reported by previous studies that age weakens the association between obesity and mortality risk–that obesity is an important risk factor for mortality for those aged 40 to 65 years, but that this risk decreases for those aged 65 years and over and may indeed provide a survival advantage.[36–38] These assertions have been challenged by Masters et al. who accounted for both the 2-way interactions between obesity and age at survey as well as cohort variation in mortality, and found a strengthening with age of the association between mortality risk and obesity.[39] It would be worthwhile to assess both the length of time lived with obesity and dynamic measures of obesity in additional studies using repeated measures collected of this cohort.

For those cases where cause of death information was available, our study found the leading cause of death was cancer, and diseases of the circulatory, respiratory and endocrine systems. Similar results were found in the overall Australian population in 2015.[14] Comparable but slightly different results can be seen in the US population in 2014: as proportions of all deaths, heart disease (males 24.5%, females 22.3%) and cancer (males 23.4%, females 22.6% of all deaths) ranked as the first and second leading causes of death respectively.[44] In our study, of the total 374 deaths, 244 (65.2%) of participants were in the ABSI Quartile 4 and another 79 (21.1%) were in Quartile 3, highlighting the association of obesity with mortality.

A major strength of our study is its use of biomedical rather than self-reported measures of obesity; the latter being shown to provide an under-estimation of weight but an over-estimation in height.[45] The inclusion of WC has been acknowledged as providing useful clinical information, particularly regarding CVD risk factors.[26,46] Android or "apple" shaped bodies have been recognised as having a stronger association with obesity-related health risks than gynoid or "pear" shaped bodies[47].

A further strength is the cohort study design which allows for investigation of important outcomes such as cause of death, as well as observations over time, pertaining to the same group of individuals. Our study was also able to provide information on biomedically measured health-related risk factors such as blood pressure, cholesterol, triglycerides and glycated haemoglobin.

There are a number of limitations in our study including the use of arbitrary cut-off points in analyses, and responder bias due to response rates and anthropometric measurement bias during the clinic visit. An examination of representativeness of the cohort was undertaken following baseline recruitment. It found that there were no significant differences between those people who had participated in the North West Adelaide Health Study and the comparison South Australian population, with regard to BMI, physical activity, current smoking status, proportions of current high blood cholesterol and high blood pressure, and overall health status.[25] While there was a decrease between the original baseline study group (n = 4056) and the analysis group for our study (n = 3311), and some resulting missing values, the proportions across the weight measures, mortality, demographics, risk factors and parental history of disease remained similar (see Table 1).

Finally, the developers of the ABSI highlighted the need for further studies to investigate whether ABSI could be used as an indicator of the effectiveness of lifestyle modification.[22] Focusing on reducing one’s WC, if possible, would lead to a lowering of the ABSI score and a subsequent reduction in mortality risk. Overall weight loss to reduce risk of developing multiple morbidities is a useful and worthwhile endeavour. From a public health perspective, it is encouraging to see evidence that adopting and more importantly maintaining healthy lifestyle choices such as not smoking, consuming a moderate amount of alcohol, undertaking regular exercising and eating the recommended levels of fruit and vegetables, can lead to a reduced HR for all-cause mortality as shown from NHANES data. Over a six-year period, those respondents who adhered to all four healthy lifestyle habits had a HR of 1.29 (95% CI 1.09–1.53) compared to 3.27 (95% CI 2.36–4.54) for those who did not undertake any of them[48].

In conclusion, ABSI is positively associated with mortality in Australian adults. Our study highlights the importance of using different measure of obesity to examine mortality risk and contributes to the growing use of ABSI as a useful predictor of mortality hazard in populations.

## Supporting information

S1 Table Primary cause of death by and within ICD10 chapters across ABSI quartiles (n = 374). (DOCX)

S2 Table Primary and secondary/subsequent causes of death ICD10 chapters for males and females (n = 374). (DOCX)
