# Supplementary material for: The association between A Body Shape Index and mortality: Results from an Australian cohort
Source: PLoS One. 2017 Jul 31;12(7):e0181244. doi: 10.1371/journal.pone.0181244 (PMC5536270; doi:10.1371/journal.pone.0181244)
Supplement: S1 Table — (DOCX) [file pone.0181244.s001.docx]

| **ICD10 CHAPTER** | **ABSI QUARTILES** | | | | |  | |
| --- | --- | --- | --- | --- | --- | --- | --- |
|  | **Q1** | **Q2** | **Q3** | | **Q4** | **Total** | |
| **Chapter I - Certain infectious and parasitic diseases (A00-B99)** |  |  |  | |  |  | |
| A047 Enterocolitis due to Clostridium difficile | 0 | 0 | 0 | | 1 | 1 | |
| A410 Sepsis due to Staphylococcus aureus | 0 | 0 | 1 | | 0 | 1 | |
| A419 Sepsis, unspecified organism | 1 | 0 | 1 | | 3 | 5 | |
| B909 Sequelae of respiratory and unspecified tuberculosis | 0 | 0 | 0 | | 1 | 1 | |
| **SUB-TOTAL** | **1** | **0** | **2** | | **5** | **8** | |
| **Chapter II Neoplasms (C00-D48)** |  |  |  | |  |  | |
| C07 Malignant neoplasm of parotid gland | 0 | 0 | 0 | | 1 | 1 | |
| C159 Malignant neoplasm of esophagus, unspecified | 0 | 0 | 2 | | 3 | 5 | |
| C169 Malignant neoplasm of stomach, unspecified | 0 | 1 | 1 | | 3 | 5 | |
| C180 Malignant neoplasm of cecum | 0 | 0 | 0 | | 2 | 2 | |
| C189 Malignant neoplasm of colon, unspecified | 0 | 1 | 1 | | 5 | 7 | |
| C19 Malignant neoplasm of rectosigmoid junction | 1 | 0 | 0 | | 0 | 1 | |
| C20 Malignant neoplasm of rectum | 0 | 0 | 0 | | 1 | 1 | |
| C220 Liver cell carcinoma | 0 | 0 | 0 | | 2 | 2 | |
| C221 Intrahepatic bile duct carcinoma | 0 | 0 | 0 | | 3 | 3 | |
| C229 Malignant neoplasm of liver, not specified as primary or secondary | 0 | 0 | 1 | | 1 | 2 | |
| C259 Malignant neoplasm of pancreas, unspecified | 1 | 2 | 1 | | 0 | 4 | |
| C260 Malignant neoplasm of intestinal tract, part unspecified | 0 | 0 | 0 | | 2 | 2 | |
| C269 Malignant neoplasm of ill-defined sites within the digestive system | 0 | 0 | 0 | | 1 | 1 | |
| C329 Malignant neoplasm of larynx, unspecified | 0 | 0 | 0 | | 1 | 1 | |
| C349 Malignant neoplasm of unspecified part of bronchus or lung | 2 | 2 | 5 | | 17 | 26 | |
| C410 Malignant neoplasm of bone and articular cartilage of other and unspecified sites | 0 | 0 | 0 | | 1 | 1 | |
| C412 Malignant neoplasm of vertebral column | 0 | 0 | 0 | | 1 | 1 | |
| C439 Malignant melanoma of skin, unspecified | 0 | 0 | 1 | | 2 | 3 | |
| C459 Mesothelioma, unspecified | 0 | 0 | 1 | | 3 | 4 | |
| C509 Malignant neoplasm of breast of unspecified site | 2 | 4 | 1 | | 0 | 7 | |
| C539 Malignant neoplasm of cervix uteri, unspecified | 1 | 0 | 0 | | 0 | 1 | |
| C549 Malignant neoplasm of corpus uteri, unspecified | 0 | 1 | 0 | | 0 | 1 | |
| C56 Malignant neoplasm of ovary | 0 | 0 | 1 | | 0 | 1 | |
| C61 Malignant neoplasm of prostate | 0 | 1 | 7 | | 8 | 16 | |
| C64 Malignant neoplasm of kidney, except renal pelvis | 0 | 1 | 0 | | 0 | 1 | |
| C679 Malignant neoplasm of bladder, unspecified | 0 | 0 | 0 | | 3 | 3 | |
| C689 Malignant neoplasm of urinary organ, unspecified | 0 | 0 | 0 | | 1 | 1 | |
| C711 Malignant neoplasm of frontal lobe | 0 | 0 | 1 | | 0 | 1 | |
| C719 Malignant neoplasm of brain, unspecified | 2 | 0 | 1 | | 4 | 7 | |
| C762 Malignant neoplasm of abdomen | 0 | 0 | 1 | | 0 | 1 | |
| C787 Secondary malignant neoplasm of liver and intrahepatic bile duct | 0 | 1 | 0 | | 0 | 1 | |
| C80 Malignant neoplasm without specification of site | 1 | 1 | 2 | | 3 | 7 | |
| C819 Hodgkin lymphoma, unspecified | 0 | 0 | 0 | | 1 | 1 | |
| C859 Non-Hodgkin lymphoma, unspecified | 0 | 0 | 2 | | 1 | 3 | |
| C900 Multiple myeloma | 0 | 0 | 1 | | 1 | 2 | |
| C911 Chronic lymphocytic leukaemia of B-cell type | 0 | 1 | 0 | | 0 | 1 | |
| C920 Acute myeloblastic leukemia | 0 | 0 | 0 | | 1 | 1 | |
| C939 Monocytic leukemia, unspecified | 0 | 0 | 1 | | 0 | 1 | |
| C97 Malignant neoplasms of independent (primary) multiple sites | 0 | 1 | 0 | | 3 | 4 | |
| D181 Lymphangioma, any site | 0 | 0 | 1 | | 0 | 1 | |
| D329 Benign neoplasm of meninges, unspecified | 0 | 0 | 0 | | 1 | 1 | |
| D361 Benign neoplasm of peripheral nerves and autonomic nervous system | 0 | 0 | 0 | | 1 | 1 | |
| D432 Neoplasm of uncertain behavior of brain, unspecified | 0 | 0 | 0 | | 1 | 1 | |
| D469 Myelodysplastic syndrome, unspecified | 0 | 0 | 0 | | 2 | 2 | |
| D471 Chronic myeloproliferative disease | 1 | 0 | 0 | | 0 | 1 | |
| **SUB-TOTAL** | **11** | **17** | **32** | | **80** | **140** | |
| **Chapter III Diseases of the blood and blood-forming organs and certain disorders involving the immune mechanism (D50-D89)** |  |  |  | |  |  | |
| D649 Anemia, unspecified | 0 | 0 | 0 | | 1 | 1 | |
| D689 Coagulation defect, unspecified | 0 | 0 | 0 | | 1 | 1 | |
| **SUB-TOTAL** | **0** | **0** | **0** | | **2** | **2** | |
| **Chapter IV Endocrine, nutritional and metabolic diseases (E00-E90)** |  |  |  | |  |  | |
| E105 Type 1 diabetes mellitus with circulatory complications | 0 | 0 | 0 | | 1 | 1 | |
| E115 Type 2 diabetes mellitus with circulatory complications | 0 | 0 | 0 | | 1 | 1 | |
| E119 Type 2 diabetes mellitus without complications | 0 | 0 | 2 | | 0 | 2 | |
| E149 Unspecified diabetes mellitus without complications | 0 | 1 | 1 | | 3 | 5 | |
| E669 Obesity, unspecified | 0 | 0 | 0 | | 2 | 2 | |
| E780 Pure hypercholesterolemia | 0 | 0 | 0 | | 1 | 1 | |
| E875 Hyperkalemia | 0 | 0 | 0 | | 1 | 1 | |
| E86 Volume depletion | 0 | 0 | 0 | | 1 | 1 | |
| **SUB-TOTAL** | **0** | **1** | **3** | | **10** | **14** | |
| **Chapter V Mental and behavioural disorders (F00-F99)** | 0 | 0 | 0 | | 1 | 1 | |
| F019 Vascular dementia, unspecified | 0 | 0 | 1 | | 0 | 1 | |
| F03 Unspecified dementia | 0 | 0 | 0 | | 3 | 3 | |
| F448 Other dissociative and conversion disorders | 0 | 0 | 0 | | 1 | 1 | |
| F102 Mental and behavioural disorders due to use of alcohol; Dependence syndrome | 0 | 0 | 0 | | 1 | 1 | |
| **SUB-TOTAL** | **0** | **0** | **1** | | **5** | **6** | |
| **Chapter VI Diseases of the nervous system (G00-G99)** |  |  |  |  | |  | |
| G20 Parkinson's disease | 1 | 0 | 0 | 1 | | 2 | |
| G309 Alzheimer's disease, unspecified | 0 | 0 | 1 | 2 | | 3 | |
| G409 Epilepsy, unspecified | 0 | 1 | 0 | 0 | | 1 | |
| **SUB-TOTAL** | **1** | **1** | **1** | **3** | | **6** | |
| **Chapter IX Diseases of the circulatory system (I00-I99)** |  |  |  |  | |  | |
| I10 Essential (primary) hypertension | 0 | 0 | 1 | 0 | | 1 |  |
| I120 Hypertensive chronic kidney disease with stage 5 chronic kidney disease or end stage renal disease | 0 | 0 | 0 | 1 | | 1 |  |
| I219 Acute myocardial infarction, unspecified | 0 | 5 | 6 | 23 | | 34 |  |
| I229 Subsequent myocardial infarction of unspecified site | 0 | 0 | 0 | 1 | | 1 |  |
| I249 Acute ischaemic heart disease, unspecified | 0 | 0 | 0 | 1 | | 1 |  |
| I251 Atherosclerotic heart disease | 1 | 3 | 3 | 8 | | 15 |  |
| I255 Ischaemic cardiomyopathy | 0 | 0 | 0 | 3 | | 3 |  |
| I258 Other forms of chronic ischaemic heart disease | 1 | 0 | 0 | 0 | | 1 |  |
| I259 Chronic ischaemic heart disease, unspecified | 1 | 0 | 7 | 11 | | 19 |  |
| I272 Other secondary pulmonary hypertension | 0 | 0 | 0 | 1 | | 1 |  |
| I340 Mitral (valve) insufficiency | 0 | 0 | 0 | 1 | | 1 |  |
| I350 Aortic (valve) stenosis | 0 | 0 | 4 | 0 | | 4 |  |
| I359 Aortic valve disorder, unspecified | 0 | 0 | 0 | 1 | | 1 |  |
| I420 Dilated cardiomyopathy | 0 | 2 | 1 | 0 | | 3 |  |
| I48 Atrial fibrillation and flutter | 0 | 0 | 2 | 1 | | 3 |  |
| I500 Congestive heart failure | 0 | 0 | 0 | 2 | | 2 |  |
| I509 Heart failure, unspecified | 0 | 1 | 0 | 4 | | 5 |  |
| I615 Intracerebral haemorrhage, intraventricular | 0 | 1 | 0 | 0 | | 1 |  |
| I619 Intracerebral haemorrhage, unspecified | 0 | 0 | 0 | 2 | | 2 |  |
| I629 Intracranial haemorrhage (non-traumatic), unspecified | 0 | 0 | 1 | 0 | | 1 |  |
| I635 Cerebral infarction due to unspecified occlusion or stenosis of cerebral arteries | 0 | 0 | 0 | 1 | | 1 |  |
| I639 Cerebral infarction, unspecified | 0 | 0 | 0 | 1 | | 1 |  |
| I64 Stroke, not specified as haemorrhage or infarction | 1 | 2 | 3 | 3 | | 9 |  |
| I671 Cerebral aneurysm, non-ruptured | 0 | 0 | 1 | 0 | | 1 |  |
| I679 Cerebrovascular disease, unspecified | 0 | 1 | 0 | 1 | | 2 |  |
| I724 Aneurysm and dissection of artery of lower extremity | 0 | 0 | 1 | 0 | | 1 |  |
| I739 Peripheral vascular disease, unspecified | 0 | 0 | 0 | 2 | | 2 |  |
| I38 Endocarditis, valve unspecified | 0 | 0 | 0 | 1 | | 1 |  |
| I609 Subarachnoid haemorrhage, unspecified | 0 | 0 | 0 | 1 | | 1 |  |
| I678 Other specified cerebrovascular diseases | 0 | 0 | 0 | 1 | | 1 |  |
| **SUB-TOTAL** | **4** | **15** | **30** | **71** | | **120** |  |
| **Chapter X Diseases of the respiratory system (J00-J99)** |  |  |  |  | |  | |
| J110 Influenza with pneumonia, virus not identified | 0 | 0 | 0 | 1 | | 1 | |
| J189 Pneumonia, unspecified | 1 | 0 | 0 | 2 | | 3 | |
| J439 Emphysema, unspecified | 0 | 0 | 2 | 2 | | 4 | |
| J448 Other specified chronic obstructive pulmonary disease | 0 | 0 | 0 | 2 | | 2 | |
| J449 Chronic obstructive pulmonary disease, unspecified | 1 | 1 | 1 | 8 | | 11 | |
| J690 Pneumonitis due to food and vomit | 0 | 0 | 2 | 1 | | 3 | |
| J841 Other interstitial pulmonary diseases with fibrosis | 0 | 0 | 1 | 2 | | 3 | |
| J849 Interstitial pulmonary disease, unspecified | 0 | 1 | 2 | 2 | | 5 | |
| J939 Pneumothorax, unspecified | 0 | 0 | 0 | 1 | | 1 | |
| J984 Other disorders of lung | 0 | 0 | 0 | 1 | | 1 | |
| **SUB-TOTAL** | **2** | **2** | **8** | **22** | | **34** | |
| **Chapter XI Diseases of the digestive system (K00-K93)** |  |  |  |  | |  | |
| K559 Vascular disorder of intestine, unspecified | 1 | 0 | 1 | 0 | | 2 | |
| K650 Acute peritonitis | 0 | 1 | 0 | 0 | | 1 | |
| K729 Hepatic failure, unspecified | 0 | 0 | 0 | 1 | | 1 | |
| K746 Other and unspecified cirrhosis of liver | 0 | 0 | 0 | 2 | | 2 | |
| K859 Acute pancreatitis, unspecified | 0 | 1 | 0 | 0 | | 1 | |
| K250 Gastric ulcer; Acute with haemorrhage | 0 | 0 | 0 | 1 | | 1 | |
| K460 Unspecified abdominal hernia with obstruction, without gangrene | 0 | 0 | 0 | 1 | | 1 | |
| **SUB-TOTAL** | **1** | **2** | **1** | **5** | | **9** | |
| **Chapter XIII Diseases of the musculoskeletal system and connective tissue (M00-M99)** |  |  |  |  | |  | |
| M313 Wegener granulomatosis | 0 | 0 | 0 | 1 | | 1 | |
| M353 Polymyalgia rheumatica | 0 | 0 | 0 | 1 | | 1 | |
| **SUB-TOTAL** | **0** | **0** | **0** | **2** | | **2** | |
| **Chapter XIV Diseases of the genitourinary system (N00-N99)** |  |  |  |  | |  | |
| N179 Acute renal failure, unspecified | 0 | 0 | 2 | 3 | | 5 | |
| N180 Chronic kidney disease | 0 | 0 | 0 | 1 | | 1 | |
| N189 Chronic kidney disease, unspecified | 0 | 0 | 1 | 1 | | 2 | |
| N19 Unspecified kidney failure | 0 | 1 | 0 | 1 | | 2 | |
| N390 Urinary tract infection, site not specified | 0 | 0 | 0 | 1 | | 1 | |
| **SUB-TOTAL** | **0** | **1** | **3** | **7** | | **11** | |
| **Chapter XVIII Symptoms, signs and abnormal clinical and laboratory findings, not elsewhere classified (R00-R99)** |  |  |  |  | |  | |
| R99 Other ill-defined and unspecified causes of mortality | 0 | 0 | 0 | 1 | | **1** | |
| **SUB-TOTAL** | **0** | **0** | **0** | **1** | | **1** | |
| **Chapter XX External causes of morbidity and mortality (V01-Y98)** |  |  |  |  | |  | |
| V031 Pedestrian injured in collision with car, pick-up truck or van; Traffic accident | 0 | 0 | 1 | 0 | | 1 | |
| V436 Car occupant injured in collision with car, pick-up truck or van; Passenger injured in traffic accident | 1 | 1 | 0 | 0 | | 2 | |
| V446 Car occupant injured in collision with heavy transport vehicle or bus; Passenger injured in traffic accident | 0 | 1 | 0 | 0 | | 1 | |
| V475 Car occupant injured in collision with fixed or stationary object; Driver injured in traffic accident | 0 | 0 | 0 | 1 | | 1 | |
| V476 Car occupant injured in collision with fixed or stationary object; Passenger injured in traffic accident | 0 | 1 | 0 | 0 | | 1 | |
| V685 Occupant of heavy transport vehicle injured in non-collision transport accident; Driver injured in traffic accident | 0 | 0 | 1 | 0 | | 1 | |
| W19 Unspecified fall | 0 | 0 | 0 | 1 | | 1 | |
| W199 Unspecified fall | 0 | 0 | 0 | 2 | | 2 | |
| X599 Exposure to unspecified factor causing other and unspecified injury | 0 | 0 | 0 | 1 | | 1 | |
| X640 Intentional self-poisoning by and exposure to other and unspecified drugs, medicaments and biological substances | 1 | 0 | 0 | 0 | | 1 | |
| X670 Intentional self-poisoning by and exposure to other gases and vapours | 0 | 0 | 0 | 1 | | 1 | |
| X70 Intentional self-harm by hanging, strangulation and suffocation | 0 | 1 | 0 | 1 | | 2 | |
| Y442 Agents primarily affecting blood constituents - Anticoagulants | 0 | 0 | 0 | 1 | | 1 | |
| V204 Motorcycle rider injured in collision with pedestrian or animal; Driver injured in traffic accident | 0 | 0 | 1 | 0 | | 1 | |
| W014 Fall on same level from slipping, tripping and stumbling | 0 | 0 | 0 | 1 | | 1 | |
| W190 Unspecified fall | 0 | 0 | 0 | 1 | | 1 | |
| W011 Fall on same level from slipping, tripping and stumbling; Residential institution | 0 | 0 | 0 | 1 | | 1 | |
| Y260 Exposure to smoke, fire and flames, undetermined intent; Home | 0 | 0 | 0 | 1 | | 1 | |
| **SUB-TOTAL** | **2** | **4** | **3** | **12** | | **21** | |
| **TOTAL** | **22** | **43** | **84** | **225** | | **374** | |
