# Supplementary material for: The association between A Body Shape Index and mortality: Results from an Australian cohort
Source: PLoS One. 2017 Jul 31;12(7):e0181244. doi: 10.1371/journal.pone.0181244 (PMC5536270; doi:10.1371/journal.pone.0181244)
Supplement: S2 Table — (DOCX) [file pone.0181244.s002.docx]

| ***ICD10 MAJOR CHAPTER*** | ***PRIMARY CAUSE OF DEATH*** | | | | ***SECONDARY/SUBSEQUENT CAUSE OF DEATH**** | | | |
| --- | --- | --- | --- | --- | --- | --- | --- | --- |
|  | ***Male*** | | ***Female*** | | ***Male (n=232)*** | | ***Female (n=142)*** | |
|  | ***n*** | ***%*** | ***n*** | ***%*** | ***n*** | ***%*** | ***n*** | ***%*** |
| Neoplasms (C00-D49) | 90 | 38.8 | 50 | 35.9 | 23 | 9.9 | 15 | 10.6 |
| Diseases of the circulatory system (I00-I99) | 74 | 32.3 | 46 | 32.4 | 92 | 39.7 | 57 | 40.1 |
| Diseases of the respiratory system (J00-J99) | 18 | 7.8 | 16 | 11.3 | 62 | 26.7 | 18 | 12.7 |
| External causes of morbidity (V00-Y99) | 12 | 4.7 | 9 | 4.9 | 56 | 24.1 | 32 | 22.5 |
| Endocrine, nutritional and metabolic diseases (E00-E89) | 10 | 4.3 | 4 | 2.8 | 20 | 8.6 | 10 | 7.0 |
| Diseases of the genitourinary system (N00-N99) | 4 | 1.7 | 7 | 4.9 | 35 | 15.1 | 11 | 7.7 |
| Diseases of the digestive system (K00-K95) | 5 | 2.2 | 4 | 2.8 | 10 | 4.3 | 8 | 5.6 |
| Certain infectious and parasitic diseases (A00-B99) | 5 | 2.2 | 3 | 2.1 | 11 | 4.7 | 9 | 6.3 |
| Diseases of the nervous system (G00-G99) | 5 | 2.2 | 1 | 0.7 | 9 | 3.9 | 5 | 3.5 |
| Mental, behavioural and neurodevelopmental disorders (F01-F99) | 4 | 1.7 | 2 | 2.1 | 10 | 4.3 | 8 | 5.6 |
| Diseases of the blood and blood-forming organs and certain disorders involving the immune mechanism (D50-D89) | 2 | 0.9 | 0 | 0.0 | 4 | 1.7 | 3 | 2.1 |
| Diseases of the musculoskeletal system and connective tissue (M00-M99) | 2 | 0.9 | 0 | 0.0 | 2 | 0.9 | 7 | 4.9 |
| Symptoms, signs and abnormal clinical and laboratory findings, not elsewhere classified (R00-R99) | 1 | 0.4 | 0 | 0.0 | 16 | 6.9 | 5 | 3.5 |
| Injury, poisoning and certain other consequences of external causes (S00-T88) | - | - | - | - | 14 | 6.0 | 7 | 4.9 |
| Diseases of the skin and subcutaneous tissue (L00-L99) | - | - | - | - | 2 | 0.9 | 2 | 1.4 |
| Diseases of the ear and mastoid process (H60-H95) | - | - | - | - | 1 | 0.4 | - | - |
| **Total** | **232** | **100.0** | **142** | **100.0** | - | - | - | - |

** Multiple causes*
